# Discovering Main Genetic Interactions with LABNet LAsso-Based Network Inference

**DOI:** 10.1371/journal.pone.0110451

**Published:** 2014-11-04

**Authors:** Francesco Gadaleta, Kristel Van Steen

**Affiliations:** Montefiore Institute University of Liege, Liege, Belgium; University of Texas School of Public Health, United States of America

## Abstract

Genome-wide association studies can potentially unravel the mechanisms behind complex traits and common genetic diseases. Despite the valuable results produced thus far, many questions remain unanswered. For instance, which specific genetic compounds are linked to the risk of the disease under investigation; what biological mechanism do they act through; or how do they interact with environmental and other external factors? The driving force of computational biology is the constantly growing amount of big data generated by high-throughput technologies. A practical framework that can deal with this abundance of information and that consent to discovering genetic associations and interactions is provided by means of networks. Unfortunately, high dimensionality, the presence of noise and the geometry of data can make the aforementioned problem extremely challenging. We propose a penalised linear regression approach that can deal with the aforementioned issues that affect genetic data. We analyse the gene expression profiles of individuals with a common trait to infer the network structure of interactions among genes. The permutation-based approach leads to more stable and reliable networks inferred from synthetic microarray data. We show that a higher number of permutations determines the number of predicted edges, improves the overall sensitivity and controls the number of false positives.

## Introduction

Any biological system is characterised by interactions between components. The study of these interactions is essential to understanding the mechanisms that regulate complex diseases and to unravel the functional aspects of genetic compounds. In several fields of research, from social to telecommunication and biology, system interactions are increasingly represented by graphical models [1]–[3]. Generally speaking, those are defined by a set of nodes and a set of edges. Each node usually represents a specific biological component that interacts with others to perform specific functions. Edges may have several meanings, depending on the type of interactions they represent, such as similarity, causality, distance, etc. In the field of network theory and genetics, the nodes of a graph usually represent genes and the edges represent the interactions among nodes. Consequently, a network graph of genetic interactions is a suitable way to visualise clusters, detect modules or pathways, according to the purpose of the analysis. Network modelling has proven to be an effective approach in computational biology due to the straight-forward representation of conditional dependency between variables [4], [5]. It is known that genes act in clusters and their individual effects tend to be characterised by a smaller magnitude within the system as a whole [6], [7]. Graphical models facilitate the detection of main genetic effects. Moreover, pathways of genes become more visible to the researcher who investigates the data, giving a more complete explanation of the biological function that the pathway itself performs. One viable way to represent the interactions of the nodes of a graph - and consequently the topology of the resulting network - is usually represented by the adjacency matrix *β* = *β_ij_*. The values of each entry (*i*, *j*) in the adjacency matrix represent the magnitude of the interaction between two nodes, whereas zeros are equivalent to absence of interaction between node *i* and node *j*. Specifically to the field of computational biology, one possible way to learn the structure of genetic interactions is to analyse the expression profile of a number of genes. The task becomes challenging due to the presence of noise in the measurements, the high dimensionality of data and multicollinearity of variables. Despite active research in the field of high-density oligonucleotide arrays, noise still represents a consistent source of error. Any analysis subsequent to the measurement of a subset of genes should take into consideration the artifacts that are usually introduced by noise or by the computational methods performed to mitigate it [8], [9]. In addition to the presence of noise, high dimensionality is a very common aspect of genetic data. The number of genes *p*, usually much larger than the number of individuals *n*, makes the task of discovering interactions extremely difficult. Without loss of generality, the problem of inferring the conditional independence between variables is equivalent to the problem of computing the sample covariance matrix of the interactions among variables. In the case of high dimensional data, as well as in a more relaxed case in which the number of individuals has a similar order of magnitude as the number of genes, the inverse of the sample covariance matrix does not exist [10]. This makes the solution of the interaction problem numerically unstable and the discovered interactions unreliable.

Finally, gene expression profiles are affected by the presence of multicollinearity [11], [12], namely two or more genes or genetic compounds can be highly correlated. Highly correlated predictor variables can give rise to non-sensical results or, specifically to regression methods, can lead to parameter estimates of incorrect magnitude and sign (*harmful multicollinearity*). Moreover, the greater the number of covariates, the higher the risk of such critical scenarios [13]. A number of techniques to mitigate the problem of multicollinearity have been indicated in the literature. Regressing each covariate on the others and investigating the stability of regression models to predicting the response variable are two methods that have been denoted in [13]. The same line of conclusion is depicted in [14], which states that successful forecast with multicollinear variables requires both a stable dependency relationship between the response and the independent variables and stable interdependency relationships within the predictors. Collecting additional data as a solution of the multicollinearity problem is suggested in [13], [14]. The presence of multicollinearity can influence the performance of methods that rely on regression. The regression coefficient of a predictor variable's importance on the target variable has the tendency to lose precision with respect to the case in which the same genes were uncorrelated. From a biological perspective, it is broadly recognised that strong genetic correlations are frequent in microarray data and that, in contrast, complete independence between any two gene expression measurements is rare [15]. Therefore, it is expected that functionally related genes are correlated to each other and might be co-expressed. This biological phenomenon can be explained by assuming the presence of high correlation for a subset of genes in the dataset under study. Moreover, as the gene sets to be tested are usually chosen on the basis of functional annotation, it should be expected that many of the tested genes might be, in fact, correlated [15]. Some regression-based methods like the one described in this paper are even more sensitive to the presence of multicollinearity as they tend to select only one or few highly correlated variables.

We propose a penalised linear regression approach that can deal with the aforementioned issues affecting genetic data. We analyse the gene expression profiles of individuals with a common trait to infer the network structure of interactions among genes. The core idea consists in reducing the number of meaningful interactions with each gene, in order to build a sparse network. Penalised linear regression (Lasso) has been investigated in seminal work reported in [16]–[19], in which each variable is considered response and the remaining ones are independent covariates. In the aforementioned work, bootstrapping has been extensively used to improve the stability of the predicted interactions. Unfortunately, the nature of genetic data and the presence of highly correlated variables can play a detrimental role that affects the overall reliability of discovered interactions. Specifically, Lasso-based regression procedures are known to deal poorly with highly correlated variables since only one in a group of multi correlated covariates is selected. Bootstrapping does not seem to mitigate such a troublesome condition.

In this paper, we consider the use of Lasso penalised regression as a starting point. We subsequently rely on a permutation-based approach in order to increase the significance of predicted interactions.

In Section Approach, we describe the method in detail. In Section Results, we measure the performance of our approach on simulated genetic networks of different size. Conclusion and future developments are drawn in the proper Sections.

## Methods

Gene expression data are usually represented by the matrix 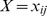 of the expression profiles of 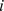 genes and 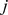 individuals or sample tissues. The main goal of the approach described in the current section is to infer the network topology that regulates the main interactions of the genes under investigation. Generally speaking, a network model is formed by a set of vertices 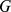, representing the genes in our specific case, and a set of edges 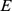 representing pairwise interactions. The existence of edge 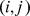 represents the conditional dependency between gene 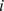 and gene 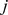. If such an edge is not present, the two genes are considered conditionally independent, in the symbolic representation 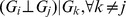. In the specific application described in this paper, we aim at finding the best set of neighbours associated to each gene. We interpret the biological meaning of genetic associations within the terms specified by regression analysis. Regressing the expression value of a gene (*response*) against the remaining ones in the dataset (*independent variables*) leads to selecting a subset of the most influential genes associated with the response. Regardless of the number of mathematical models that have been considered for inferring the association between variables in genetics, linear regression is a type of analysis that has found large consensus in the field of computational biology due to its simplicity of modelling [20], [21]. One limitation of linear regression methods prevails in assuming a linear dependency between variables, a hypothesis that does not always apply in biology. One strategy to overcome such a limitation consists of splitting the problem of learning the topology of the entire network of genes into a number of smaller linear problems. This can be achieved by regressing each covariate against all the remaining ones. Such a strategy, which has been used first in the work reported in [17] makes the assumption of linearity more suitable to the analysis of biological data. Assuming the presence of linearities on a local scale is a much more convincing and appropriate conjecture that might find an application to data from genomics and proteomics. Another limitation that researchers have to take into account appears in the case of high-dimensional data. In such a scenario, the number of genes is usually some orders of magnitude larger than the number of the individuals. Penalised regression has been considered as a way to circumvent such limitation due to the presence of a penalty factor that encourages sparsity of the final network. Specifically, Lasso is one such regression method that converts the problem of estimating the covariance matrix into an optimisation problem in which a convex function, applied to each variable, is minimised.

Given 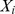 the expression of gene 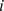 and the expression profiles of the remaining genes (referred to as 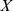, for simplicity), the Lasso-based estimate consists of providing a solution for Equation 1

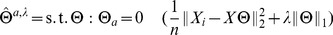
(1)


The vector of regression coefficients 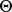 determines the conditional independence structure between variables. The 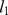-norm of the coefficient vector tends to shrink the coefficients of some variables to zero, removing them from the set of selected variables associated to the response, as extensively explained in [16]. The right choice of the shrinkage factor 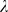 is crucial to controlling the rate of false positives and false negatives. Regardless of the number of approaches to approximate the optimal 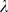, reported in [22]–[24], a reliable estimate that is widely used in practice is provided by cross-validation [25]. We use a 3-fold cross validation approach and estimate 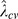 from a subset of the data. Cross-validation can be a time consuming task especially when applied to datasets with a high number of covariates. Therefore, we estimate the shrinkage factor that minimises the expected generalisation error, for a grid of 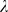 values, on the 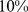 of the total number of genes. The R package 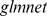 has been used to provide such an estimate.

The method we describe in this paper is a two-step approach that recursively performs the regression of Equation 1 for each gene, considered as response, with respect to all remaining genes, considered as independent variables. The response gene is not included in the set of independent variables. Regardless of biological evidence that supports the existence of self interactions and positive/negative feedback loops within regulatory networks [26]–[28], those are not considered here, in order to avoid complex interactions and simplify as much as possible the inferred network topology.

**In step 1**, the set 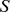 of variables associated with the current response gene is selected. We use a Lasso method that does not fit the intercept. As explained, the choice of the optimal 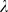 occurs prior to this stage.

**In step 2**, we use a permutation-based approach to assess the significance of the associated edges detected in step 1. The values of the response variables are permuted a number of times specified as parameter. For each permutation we count how many times each variable within the set 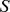 of selected genes has been selected again. At the end of the permutation test, the variables with the smallest counter are selected as the best candidate variables associated with the current response gene.

This approach is supported by the fact that after permuting the response variable, the genes selected at step 1 should be no longer associated and therefore should be considered as selected by chance.

The procedure we propose is summarised in Table 1. It selects the 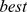 number of genes associated to the current response. Namely, the vector of the associated genes is sorted in decreasing order and the first 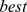 are selected (*line 5*). The parameter 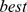 can be tuned in order to select a variable number of strong genetic effects according to the type of disease under investigation and the dataset at the researcher's disposal which, in turn, might determine the amount of significant genetic compounds to be considered for further analysis. At each permutation, the counters of the selected variables are updated (*line 9*) and after 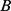 permutations the first 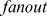 genes are selected. These variables represent the most stable genes associated with the response variable (*line 12*).

**Table 1:** Algorithm of the variable selection and permutation-based stability test.

1: **procedure** lasso2net( 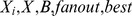 )
2: 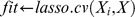
3: 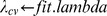
4: 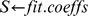
5: 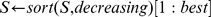
6: **while** 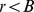 **do**
7: 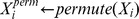
8: 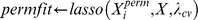
9: 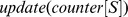 update counters of selected variables
10: 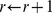
11: 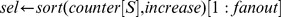 order and select first fanout
12: **return** 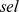

For large values of 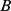 we perform an additional significance test for the smallest counter. A critical case to deal with occurs whenever different covariates are selected with similar frequency. This phenomenon in turns produces uniform values of counters for a high number of selected variables. Lasso-based regression methods are affected by issues of this type due to the fact that one from a group of highly correlated variables can be randomly selected at each permutation. To mitigate such side effects, we compute the empirical distribution of the counters of the selected covariates regressed against each permuted response. The p-value of the smallest counter is calculated from the aforementioned empirical distribution. We found that a significance level of 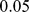 improves the precision (calculated as 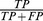) by 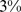.

The algorithm described above finds a solution of Equation 1 for each response variable. Subsequently, it finds the most stable non-zero regression coefficients associated to each gene. Consequently, when the described procedure is performed on the entire set of genes, an adjacency matrix can be built directly from the counters of selected variables. The aforementioned adjacency matrix can be used to visualise the network topology of the inferred network of interactions. Since we are interested in discovering genetic interactions we convert the non-zero values of the adjacency matrix to 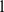, in order to denote the presence of an edge in the graph. As one would expect, the method does not guarantee the adjacency matrix to be symmetric. A symmetrisation procedure would be required before further analysis or visualisation of the predicted network.

A number of approaches that perform matrix symmetrisation have been proposed in [29]. Given two nodes 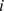 and 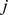 and the weights of the edges 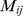 and 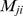, the symmetric adjacency matrix can be built by taking the average value as in 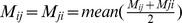; by selecting the largest weight 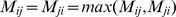; or by selecting the smallest weight 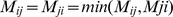. For a binary adjacency matrix, in which each entry represents the presence or absence of the edge 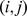, the AND rule will set 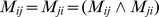. In order to detect a generic association between nodes 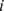 and 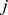, we symmetrise the adjacency matrix by applying the 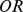 rule which considers two variables as associated if only one of the two variables is associated with the other. Namely, 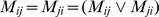.

The main goal of the work described here is to detect the structure of the network of the main genetic associations, passing over the magnitude of interaction and its direction.

## Results

In order to evaluate the performance of the method described in the Methods section, we need to compare the predicted network to the real network that generated the data. In real biological applications this procedure is usually not possible, due to the fact that the real network is, in fact, unknown. In the specific case described thus far, we take advantage of synthetic data that make such a performance evaluation practical.

Since our algorithm is designed to analyse gene expression profiles, we generate synthetic microarray data with the Gene Net Weaver software package (GNW) [30]. The aim of GNW is to generate in-silico networks extracting modules from biological networks. These networks are simulated to produce gene expression data (steady states or time series) [31]. The aforementioned framework can be used to evaluate the performance of our inference method by comparing the predicted network with the golden standard network that generated the dataset.

We perform the approach described above on synthetic microarray data generated from simulated networks of 50 and 200 nodes. The parameters used in our experiments are summarised in Table 2.

**Table 2:** Parameters of LABnet for both 50-node and 200-node networks.

**cross-validation**	3-fold on 10% genes
**best**	80% genes
**fanout**	1
**B**	0–500

**Figure 1:**
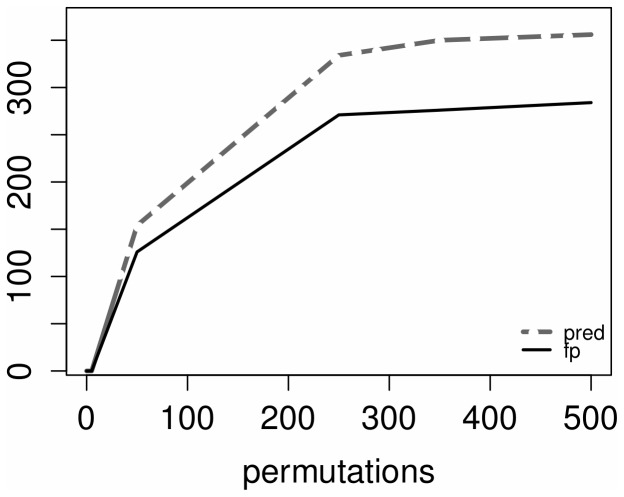
Number of predicted edges and false positives vs. number of permutations.

A set of networks has been inferred with an increasing number of permutations. One important characteristic that arises from our experiments consists in the fact that by increasing the number of permutations, the connectivity of the network is increased proportionally (Figure 1). Within the same figure it is shown that the number of false positives is limited regardless the number of predicted edges and permutations. We measure the connectivity of the network by counting the number of the predicted edges.

**Figure 2:**
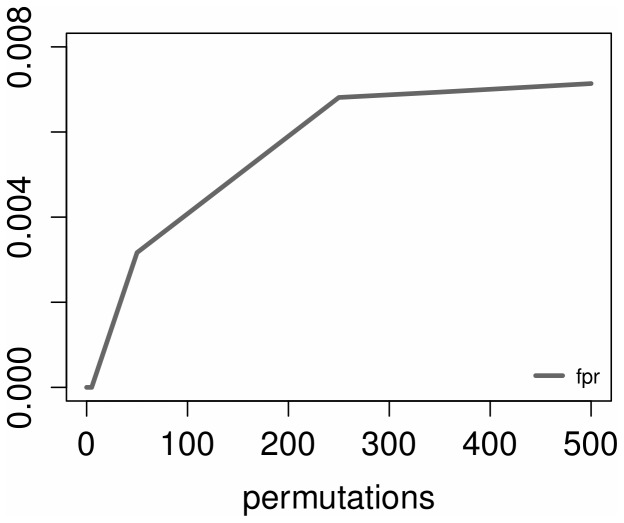
False positive rate vs. number of permutations.

**Figure 3:**
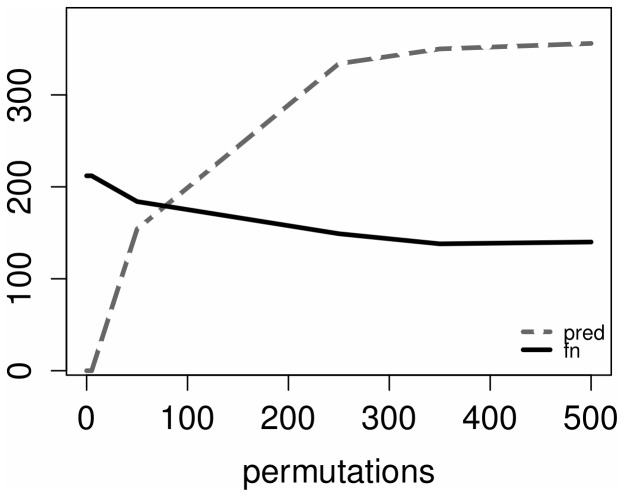
Number of predicted edges and false negatives vs. number of permutations.

In Figure 2 the false positive rate, usually referred to as accuracy, is not affected by the number of permutations but by the number of predicted edges which increases accordingly (as shown in Figure 3).

Moreover, by increasing the number of permutations the false negatives, or missed edges, tend to decrease (Figure 2). Since higher connected networks are usually affected by an increasing number of false negatives, we consider the method described above a promising approach with potential benefits to the analysis of large genetic networks.

**Figure 4:**
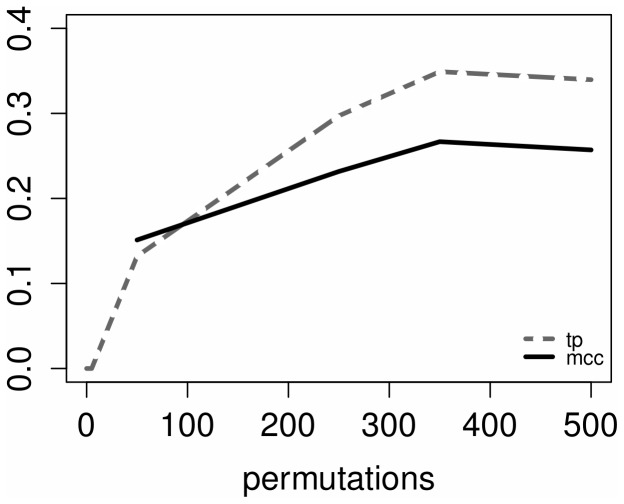
True positives and Matthew Correlation Coefficient vs. number of permutations.

We also found that the true positive rate follows the same trend of the number of permutations (Figure 4). Within the same figure the Matthew Correlation Coefficient (MCC) is also reported. The MCC is a correlation coefficient between the observed and the predicted classification (presence or absence of edges). It returns a value in the range 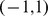, where 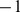 indicates total disagreement between prediction and observations, 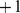 indicates perfect prediction and 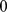 no better than random guessing. One important property of the MCC is that it takes into account the number of true negatives and true positives of the predicted network within the normalisation factor. This leads to more meaningful interpretations of the final MCC score. Biological networks are usually sparse. Therefore, prediction methods performed on such networks usually return high numbers of true negatives (absent interactions are correctly predicted). In the extreme case of empty predicted network (a network without any edge), the number of true negatives would positively impact the overall performance of the method. It comes without saying that measuring the number of true negatives would be too optimistic. The MCC mitigates extreme cases of this type. The empty network would have a 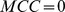.

The MCC, as introduced in [32], is calculated as
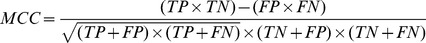
(2)


In order to compare the predicted network to the golden standard using a measure that takes into account the global structures of the graphs, two global measures have been provided, such as the degree correlation 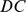 and the betweenness correlation 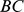.


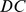
 is the correlation between the vector of the degrees of all genes in the real network and those of the predicted network. It is calculated as
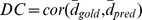
where 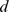 is the 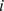-dimensional vector containing the degree of each gene.

Similarly, the betweenness correlation 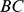 is the correlation between the same two vectors where the degree has been replaced by the betweenness centrality measure.


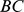
 is calculated as
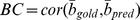
(3)where 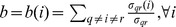, 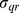 is the total number of shortest paths from node 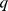 to node 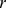 and 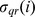 is the number of shortest paths from 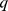 to 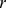 that pass through gene 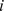.

Betweenness centrality is, in our opinion, more helpful than simple connectivity. This measure is a direct indicator of how connected the node is and its importance with respect to the global network topology.

**Figure 5:**
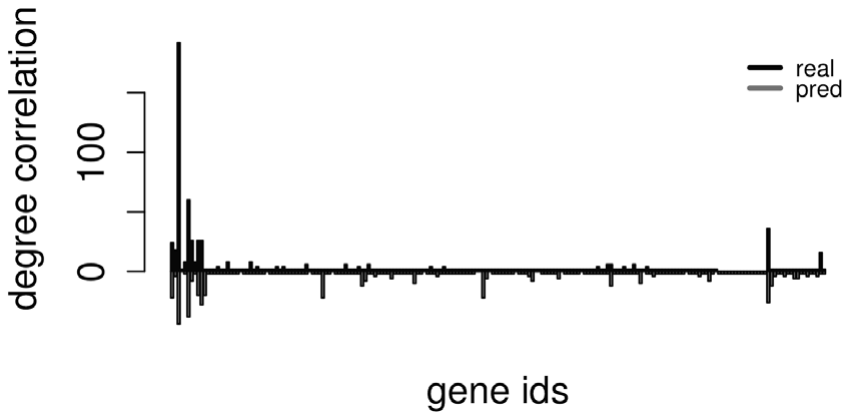
Degree correlation across real and predicted nodes.

**Figure 6:**
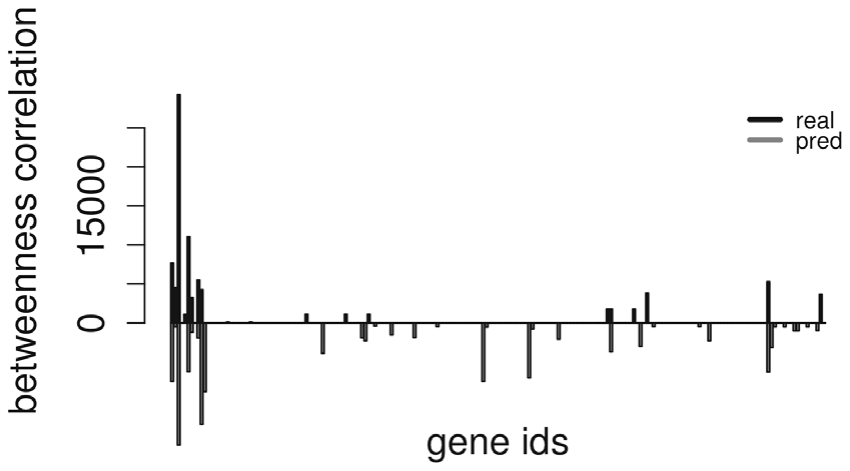
Betweenness correlation across real and predicted nodes.

As it can be seen in Figure 5 and Figure 6 there is a strong degree correlation (0.83) and betweenness correlation (0.86) between the nodes of the predicted and real networks. The two measures and the aforementioned strong correlations support the evidence that the topology of the real network is conserved within the predicted network, following the same power law degree distribution of the original network that generated the data. Due to the fact that GNW generates network from real life templates, we expect similar results in real biological data.

### Performance

We implemented LABNet in R and we used the *glmnet* package to perform both the Lasso penalty estimation and variable selection. For each gene (node) the algorithm performs a number of permutations to compute the most significant variables associated to the current gene. Therefore, the code that performs such permutations is the most demanding in terms of computing resources and time. The permutation-based variable selection described in the previous section can be performed independently from the rest of the code. As a consequence, the bottleneck of our method seems to be ideally suited for parallel architectures. We implemented a parallel version that consistently improves the overall performance of the algorithm, with respect to its sequential execution. The response permutation code has been written to perform within the *snowfall* environment, distributed as R package in CRAN.

A summary of the performance improvement achieved by parallel code is provided in Table 3. Moreover, we found that the speedup is more consistent as the number of permutations is increased.

**Table 3:** Timings for 4 different execution of LABNet running in sequential (1 CPU) and parallel environments from 2 to 4 CPUs on general purpose hardware (1.3 GHz Intel Core i5), 4GB RAM.

Genes	Perm	1 CPU	2 CPU	3 CPU	4 CPU
50	500	269	175	153	135
50	1000	547	347	282	276
200	500	2846	2073	1997	1942

*Perm* indicates the number of permutations per gene.

In a setting with 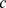 computing nodes, 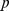 genes, 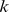 permutations per gene, 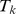 seconds required to perform 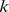 permutations on one computing node, the speedup introduced by the parallel approach can be calculated as 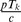. In the aforementioned formula, the time needed to perform the first selection of covariates and set the computing nodes within the cluster are not counted. Therefore, the expression will indicate better performance than the one we measure effectively in our experiments. As the number of available computing nodes approaches the number of covariates, the total time required by our algorithm is determined by the number of permutations performed on the single computing node.

## Discussion

The Lasso-based procedure performed on microarray data is enhanced by a permutation approach that consistently improves the stability of the inferred network structure. The purpose of permuting the response variable is to break the link with the other independent variables by optimising an equivalent convex function which selects a number of variables close to those selected for the original (not permuted) response. Since the permutation affects only the response gene, the structure of the permuted data is equivalent to the original one. Moreover, using the same 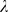 increases the speed of the algorithm due to the fact that cross-validation is no longer required. Results from simulated genetic data are encouraging and consent to perform our approach to predict genetic interactions from real biological datasets. However, we address some limitations we intend to investigate in the near future.

As already stated, genetic data are usually affected by measurement noise and high number of variables collected from different datasets such as gene expression profiles, SNPs, methylation and clinical data.

The curse of dimensionality can set a limit on the number of permutations to perform. Due to the fact that our method relies on permuting each response variable in order to increase the stability of the discovered interactions, the overall performance is directly affected by the total number of genes in the dataset.

The variable selection procedure consistently depends on the value of the shrinkage factor 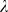, estimated on a subset of the covariates. Obviously, it might occur a prior exclusion of significant genes from further analyses in the case of a too restrictive shrinkage factor. An alleviation to this risk (which can directly determine the false negative rate) consists in replacing the pure Lasso penalty with an elastic net procedure of the type
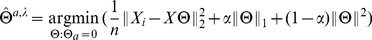
(4)


In such a scenario it would be necessary to estimate an additional parameter 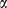. To the other extreme, a pure ridge-regression procedure would not benefit from the permutation-based stability test, due to the fact that ridge-regression procedures tend to include all the covariates in the model. Moreover, our method ignores the value of the regression coefficients and selects a subset of genes with the best permutation score. In a ridge-regression setting all covariates would be selected an equal number of times.

Another aspect we intend to probe regards the direction of the interactions. In our analysis we ignore the direction of each edge in the graph. A relaxation of the problem of learning the network topology consists in considering the interaction 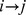 equivalent to the interaction 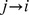. Although this simplification makes the construction of the overall network consistently easier, it might lead to inconsistencies from a biological perspective. As a matter of fact, gene regulations are known to have a direction, usually referred to as activation and inhibition. Activation and inhibition are essential regulatory mechanisms in the transcriptional machinery of the cell and are causes for up- and down-regulation of particular genes [33].

Learning the directionality of network edges represents an additional complexity that is plausible to deal with only in the presence of a large number of samples, or by integrating complementary data sources of known interactions. Therefore, the need for integrating different data sources is twofold: data integration can increase the stability of all discovered interactions and their direction and, specifically to our method, it can reduce the number of required permutations per gene. We believe that data integration can consistently improve the overall performance of the described approach.

We endorse our approach to be deployed in a data analysis pipeline in order to 1) analyse different data sources 2) build the local network from each dataset 3) increase the stability of predicted interactions by permutation and 4) integrate each singular network into a more stable and complete graph. We are currently extending our network inference method to implement the aforementioned data analysis pipeline.

## Conclusion

We presented LABNet, a Lasso-based approach to detect main genetic interactions from gene expression profiles. Penalised regression in concert with a permutation-based procedure determines whether the predicted interactions are stable across experiments. The higher number of permutations not only improves the sensitivity of the method by reducing the number of false negatives, but it also determines the overall number of predicted edges. This does not seem to affect consistently the false positive rate. Due to the features that we have described and the promising results on synthetic data, the approach is a good candidate to further investigate and expand for the analysis of heterogeneous sources of genetic data.
